# Purification and Characterization of Shiga Toxin 2f, an Immunologically Unrelated Subtype of Shiga Toxin 2

**DOI:** 10.1371/journal.pone.0059760

**Published:** 2013-03-26

**Authors:** Craig Skinner, Stephanie McMahon, Reuven Rasooly, John Mark Carter, Xiaohua He

**Affiliations:** Western Regional Research Center, United States Department of Agriculture, Agricultural Research Service, Albany, California, United States of America; Centre National de la Recherche Scientifique, Aix-Marseille Université, France

## Abstract

**Background:**

Shiga-like toxin 2 (Stx2) is one of the most important virulence factors in enterohaemorrhagic Escherichia coli (E. coli) strains such as O157H7. Subtypes of Stx2 are diverse with respect to their sequence, toxicity, and distribution. The most diverse Stx2 subtype, Stx2f, is difficult to detect immunologically, but is becoming more frequently associated with human illness.

**Methods and Findings:**

A purification regimen was developed for the purification of Stx2f involving cation exchange, hydrophobic interaction, anion exchange, and gel filtration. The molecular weight of Stx2f B-subunit was approximately 5 kDa, which appeared significantly smaller than that of Stx2a (6 kDa) on a SDS-PAGE gel, although the size of the A subunit was similar to Stx2a (30 kDa). Stx2f was shown to be active in both cell-free and cell-based assays. The 50% cytotoxic dose in Vero cells was 3.4 or 1.7 pg (depending on the assay conditions), about 3–5 times higher than the archetypical Stx2a, while the activity of Stx2f and Stx2a in a cell-free rabbit reticulocyte system was similar. Stx2f bound to both globotriose-lipopolysaccharide (Gb3-LPS) and globotetraose-LPS (Gb4-LPS, mimics for globotriaosylceramide and globotetraosylceramide, respectively), but its ability to bind Gb4-LPS was much stronger than Stx2a. Stx2f was also much more stable at low pH and high temperature compared to Stx2a, suggesting the toxin itself may survive harsher food preparation practices.

**Conclusions:**

Here, we detail the purification, biochemical properties, and toxicity of Stx2f, from an *E. coli* strain isolated from a feral pigeon. Information obtained in this study will be valuable for characterizing Stx2f and explaining the differences of Stx2a and Stx2f in host specificity and cytotoxicity.

## Introduction

A group of major and potentially deadly bacterial contaminants, enterohemorrhagic *Escherichia coli* (*E. coli)* subtypes are responsible for many recent food illness outbreaks. A subset of these pathogens, Shiga toxin-producing *E. coli* (STEC), can generate symptoms from bloody diarrhea to the potentially life-threatening hemolytic uremic syndrome (HUS) [1]. The most well-known serotype of STEC is *E. coli* O157:H7, but many non-O157 serotypes can result in severe disease and food contamination outbreaks, including the deadly 2011 outbreak of O104:H4 in Germany [2]–[4]. STEC strains derive much of their virulence from the release of Shiga-like toxins (Stxs), of which there are many distinct variants and subtypes [5], [6]. The proliferation of non-O157 serotypes producing Stx suggests that STEC are becoming a very diverse and heterogeneous group of pathogens [7]. Stxs themselves appear to be growing in diversity as well, highlighting the importance of continually improving methods for their detection and characterization.

Stxs consist of a catalytic A subunit with a pentameric B subunit protein complex for targeting and binding the host receptor. They therefore fall into the AB_5_ category of bacterial toxins, along with cholera and pertussis toxins [8]. Internalization of Stx into target cells is mediated by the B-subunit pentamer, which attaches to the membrane lipid globotriaosylceramide (Gb3Cer) or globotetraosylceramide (Gb4Cer) [9]. Much of the toxicity of Stx is mediated by the catalytic A subunit, which is an rRNA *N-*glycosidase that cleaves a single adenosine residue from the 28S rRNA and inactivates 28S ribosomal subunits [10]. The two main classes of Stxs in *E. coli* are Stx1, which are almost identical to the toxin from the *Shigella* genus, and Stx2. Stx1 and Stx2 can be found independently or together in O157:H7 and other serotypes of STEC [11]. The sequences of Stxs are typically carried on a lambdoid bacteriophage, making them easily transduced not only between different serotypes of *E. coli* but also to non-pathogenic *E. coli* in infected intestines [12]. Production of Stx2 is initiated by a late-phase phage promoter, and release of the toxin occurs in part when the *E. coli* is lysed by the phage [9], [13]. Antibiotics which induce the bacterial SOS response, such as mitomycin C and ciprofloxacin, cause lysis of the STEC by bacteriophage, and therefore liberate considerable amounts of toxin [14], [15]. Late-phase phage promoter-driven genes such as Stx2 are also responsive to the SOS response. For these reasons, the prognosis for STEC-infected individuals that have been treated with certain antibiotics (e.g. ciprofloxacin) is actually worse than for those who received no antibiotic treatment at all [16].

Although any Stx can exacerbate *E. coli*-associated gastroenteritis, there are vast differences in toxicity among Stx variants and subtypes. The nomenclature recently proposed by Scheutz *et al* in 2012 was used in this study [17]. Stx2a, 2c, and 2d are associated with the development of HUS, and have much more severe clinical consequences than Stx1 [18]. Although Stx1 has been shown to be more toxic to Vero (green monkey kidney) cells than Stx2a, Stx2a is 100 times more toxic to mice than Stx1 [18], [19], perhaps explaining its increased toxicity in humans. Stx1 and Stx2a also have different toxicities upon different cell types: Stx1 is more (10 times) toxic than Stx2a to human brain macrovascular endothelial cells, while Stx2a is much more lethal (1000 times) to microvascular endothelial cells [20]. STEC strains which possess more than one type of Stx, however, may have mitigated toxicity. It has been suggested that strains that express both Stx1 and Stx2a are less toxic than those that express only Stx2, presumably due to competition for the same host cell receptors [21], although differences in serotype or host background could also account for this. Associations between different Stx2 subtypes are unknown. Stx2c and 2g are very similar to the prototypical Stx2a, whereas Stx2e is more divergent at both the genomic and amino acid level. Stx2e is found in human STEC strains, but generally only causes mild gastroenteritis [22]. In pigs, however, Stx2e production can result in deadly edema disease [23]. Stx2f is the most divergent (by genomic and amino acid sequence) of the known Stx2 subtypes. It was first isolated in pigeons [24] and initially thought to be uninvolved in human illness. However, recent studies and advancements in Stx2f detection have determined that the presence of Stx2f in human STEC is on the rise [25]. This emphasizes the need to have pure, or at least partially pure, Stx2f for developing Stx2f-specific antibodies for immunodiagnosis and investigating the role of Stx2f in the pathogenesis of human diseases.

While production and purification of recombinant Stx and its subunits have been well-documented, there are additional benefits to obtaining naturally-released toxin directly from wild type bacterial cultures. If there are any modifications that occur to the toxin while it exits the *E. coli*, these would be preserved. Any other bacterial factors that associate with Stx may also persist during a purification regimen. Purification of the prototype Stx2a and closely-related Stx2c, Stx2d, and Stx2g subtypes have been previously demonstrated [26], but purification and characterization of the Stx2e and 2f subtypes remains unreported. Our previous study demonstrates that most antibodies against Stx2a do not cross-react with Stx2e and 2f [26], and currently no effective antibodies against these subtypes are available. Purifying Stx2e and Stx2f is complicated due to the lack of tracing tools. In this study, we detail a four-step purification scheme for the Stx2f subtype and compare the properties of Stx2f with its better understood relative Stx2a. It is our expectation that obtaining pure Stx2f can pave the way toward producing Stx2f-specific antibodies, and development of improved detection methods and treatments for diseases caused by Stx2f-expressing STEC.

## Materials and Methods

### *E. coli* Strains and Growth Conditions

All strains of *E. coli* used are included in Table 1. Stx2a and Stx2f-expressing strains RM10638 (Stx2a) and RM7007 (Stx2f) were used for toxin production [26]. 20 mL starter cultures containing LB broth (Fisher) were grown overnight at 37°C with agitation, then diluted 1/50 in 500 mL LB supplemented with 50 ng/mL mitomycin C (Sigma-Aldrich) when indicated. Bacteria were then grown for 24 hours at 37°C with agitation, then centrifuged at 5 kG for 15 min. Cell pellets were autoclaved, bleached, and discarded, and the medium was sterile filtered (PVDF, 0.2 µm). The K12 *E. coli* strain was used as a non-toxin-producing control for the growth curve.

**Table 1:** *E. coli* strains used in this study.

Strain	Other names	Serotype	Biomolecule expressed	Origin^a^	Reference
RM10638		O157:H7	*stx2a*	Cow (2009)	[26]
RM7007	T4/97	O128:H2	*stx2f*	Feral pigeon	[24]
RM5034	K12				[2]
CWG308 pJCP-Gb3			Gb3-LPS (globotriose-LPS)		[27]
CWG308 pJCP-*lgt*CDE			Gb4-LPS (globotetraose-LPS)		[28]
CWG308					[27]
TOP10					Invitrogen
TOP10 pTrcHis2-Stx2fA			*stx2f* A subunit		This study

a Year of sample collection is shown in parenthesis.

### Ammonium Sulfate Precipitation

Stx2a was precipitated from 250 mL of cell supernatant by adding solid NH_4_SO_4_ to 70% saturation. The mixture was then centrifuged (5 kG, 15 min. at 4°C), and the pellet was resuspended in phosphate buffered saline (PBS, 20 mM NaPO_4_, 150 mM NaCl, pH 7.4). The Stx2a-containing pellet was then buffer exchanged using a 4 mL Zeba desalting column (Thermo Scientific) to 50 mM sodium acetate (NaOAc) pH 4.3 in preparation of cation exchange chromatography.

### pH Fixing and Cation Exchange Chromatography (CEC)

Stx2f culture supernatant pH was set to 4 using glacial acetic acid (approximately 10 mL of 14.3 M acetic acid), then the mixture was centrifuged (5 kG, 15 min.) and sterile filtered (PVDF, 0.2 µm) for clarity. This pH 4 cell supernatant was added directly to a 5 mL SP-HP cation exchange column on the Äkta FPLC (GE Life Sciences) that was previously equilibrated with 50 mM sodium acetate (NaOAc), pH 5. After a wash with 50 mM NaOAc, pH 5, toxin was eluted with a 20 mL NaCl gradient (0 to 0.6 M) in 50 mM NaOAc, pH 5.3, at 3 mL/min, and collected in 1 mL fractions. Stx2a protein was purified in the same manner, but without pH-fixing and using 50 mM NaOAc, pH 4.3 instead of pH 5.

### Hydrophobic Interaction Chromatography (HIC)

Based on ELISA results, fractions from CEC containing Stx2f were pooled, and buffer was exchanged to 50 mM sodium phosphate (NaPO_4_) +1 M NH_4_SO_4_, pH 7 using a 4 mL Zeba desalting column. Approximately 3 mL of partially (cation exchange) purified toxin was thus injected onto a 5 mL Phenyl HP column, equilibrated with 50 mM NaPO_4_+1 M NH_4_SO_4_, pH 7, on the Äkta FPLC. Toxin was then eluted with a 25 mL NH_4_SO_4_ gradient (1 to 0 M) in 50 mM NaPO_4_, pH 7, at 3 mL/min, and collected in 1 mL fractions.

### Anion Exchange Chromatography (AEC)

Based on ELISA results, fractions from HIC containing Stx2f were pooled, and buffer was exchanged to 20 mM 1,3-diamino-propane (13DAP), pH 9, using a 4 mL Zeba desalting column. Approximately 3 mL were loaded on a 1 mL Q HP anion exchange column, equilibrated with 20 mM 13DAP. Toxin was eluted with a 10 mLNaCl gradient (0 to 1 M) in 20 mM 13DAP, pH 9, at 1 mL/min, and collected in 0.5 mL fractions.

### Gel Filtration

Based on ELISA results, fractions from AEC containing Stx2f were pooled, and approximately 0.7 mL was injected onto a Sephacryl 100 16/60 gel filtration column pre-equilibrated with PBS on the Äkta FPLC. Column was eluted at 0.5 mL/min. Fractions containing pure toxin (by Coomassie staining) were collected, concentrated by an Amicon Ultra filter (10 kD pore size) (Millipore), and sterilized by a 0.2 µm PVDF filter.

### SDS-Polyacrylamide Gel Electrophoresis (SDS-PAGE), Coomassie Staining, and Western Blots

All samples for SDS-PAGE analysis were incubated for 5 min at 75°C prior to loading onto the gel, with the exception of the cell pellets (95°C for 10 min.). Gels (4%–12% NuPAGE Novex Bis-Tris mini gels from Invitrogen) were run following the manufacturer’s specifications and stained with SimplyBlue Safe Stain (Invitrogen) for visualization of proteins. For Western blots, after PAGE, the proteins were electroblotted to a PVDF membrane (pore size, 0.45 µm; Amersham Hybond-P). The membrane was blocked with 2% ECL Prime blocking agent (GE Healthcare) in PBS-Tween-20 (0.05%) (PBST). Stx’s on the membrane were detected with either mouse anti-Stx2f antibodies (prepared as indicated below) and anti-mouse IgG-HRP (Promega), or rabbit anti-Stx2a-HRP conjugate (generated from antibodies prepared as below, with the Lightning-Link HRP Conjugation Kit; Innova Biosciences) and then developed with Lumigen TMA-6 (Lumigen) substrate. Western blots were visualized with a 2 minute exposure using a FluorChem HD2 (Alpha Innotech).

### Production of Polyclonal α-Stx2f and α-Stx2a Sera

To develop the Stx2f polyclonal antibody, a His-tagged Stx2f A subunit construct was developed using the pTrcHis2 TOPO cloning kit (Invitrogen). His-tagged Stx2f A subunit expressed in TOP10 cells (Invitrogen) was induced with 1 mM IPTG and purified using a Ni-NTA affinity column (Qiagen). 5 µg of Stx2f in 2 mL PBS was used to reconstitute a vial of MPL-TDM (Sigma) adjuvant and injected intraperitoneally into a Balb/c mouse 3 times at 2 week intervals. One week after the 3^rd^ injection, sera were collected using a tail vein bleed. To develop a Stx2a polyclonal antibody, a rabbit was immunized with catalytically inactive Stx2a toxoid (E167Q mutation) and serum was collected by cardiac puncture with exsanguination (procedures performed by Pacific Immunology).

### Ethics Statement

All procedures with animals were carried out according to institutional guidelines for husbandry approved by the Animal Care and Use Committee of the U.S. Department of Agriculture, Western Regional Research Center (USDA ACUC Protocol #09-J-10).

### Molecular Weight and Isoelectric Point Calculation

Estimated molecular weights and isoelectric points (pI) were calculated using the ExPASy Compute pI/MW tool (http://web.expasy.org/compute_pi/). When calculating the pI of the toxin complex, the amino acid sequence of the A subunit followed by five repeats of the B subunit sequence were used as the input.

### ELISAs

ELISAs for monitoring Stx2a and 2f purifications were direct-well binding assays. Fractions collected were diluted 1/25 in a final volume of 100 µL in PBS and bound directly to a flat-well Nunc maxisorp plate overnight at 4°C. After blocking the plates with 200 µl 5% milk in PBS-Tween 20 (0.05%) (PBST), they were then incubated with 100 µl primary antibody: Sifin 2A (clone VT135/6-B9, Sifin Institute, Berlin, Germany) for Stx2a; mouse polyclonal serum for Stx2f. Both antibodies were diluted 1/10,000 in blocking buffer for 1 hour at RT. Goat anti-mouse IgG-HRP (Promega) was used as a secondary antibody, and the plates were developed with TMB substrate (Pierce), followed by addition of 100 µl 0.3 N H_2_SO_4_. Absorbance was measured at 450 nm on a Victor II plate reader (Perkin Elmer). Between each step plates were washed 3 times with 200 µl PBST.

### Gb3-LPS and Gb4-LPS Binding Assays

The Gb3/Gb4-LPS binding assay was conducted similar to a traditional sandwich ELISA. Formaldehyde-fixed *E. coli* cells expressing globotriose-lipopolysaccharide (Pk glycan, referred to in this study as “Gb3-LPS”, a Gb3 mimic) or globotetraose-LPS (P glycan, referred to in this study as “Gb4-LPS”, a Gb4 mimic) or control *E. coli* cells (CWG308 pJCP-Gb3, CWG308 pJCP-lgtCDE, and CWG308 ctrl, respectively) [27], [28] were diluted to 0.05 OD_600_ in carbonate buffer (0.1 M NaCO_3_, pH 9.6) and 100 µl was bound to the wells of a black 96-well Nunc Maxisorp plate by incubating at 50°C until all liquid had evaporated. Wells were then blocked with 200 µl 5% milk/PBST for 1 hour at RT. Stx2f or Stx2a toxin was then diluted in blocking buffer to the indicated concentrations and incubated on the plate for 1 hour at RT. Anti-Stx2f mouse or anti-Stx2a rabbit polyclonal serum was then added at a 1/5,000 dilution in blocking buffer and incubated for 1 hour at RT. Goat anti-mouse IgG HRP or goat anti-rabbit IgG HRP (Promega) was then added (100 µl at 1/5,000) and incubated for 1 hour at RT. Signal was detected with 100 µl SuperSignal West Pico Chemiluminescent Substrate (Thermo Scientific) and read on a Victor II plate reader (Perkin Elmer). Between each step plates were washed 3 times with 200 µl PBST.

### *In vitro* Translation Assay

Cell-free translation assays were conducted with a previously described rabbit reticulocyte lysate protocol [29], using various dilutions of Stx2f and Stx2a to determine 50% inhibition.

### Extinction Coefficient Calculation

The absorbance of pure Stx2f at 280 nm at 200, 100, and 50 µg/mL was analyzed by Nanodrop (Thermo Scientific) in triplicate. These values were plotted and, using a linear standard curve, extrapolated to estimate an extinction coefficient for Stx2f at 1 mg/mL.

### Vero Cell Cytotoxicity Assays

Vero (African green monkey kidney) cells [30] were cultured in Dulbecco’s Modified Eagle Medium (DMEM, Invitrogen) supplemented with 10% fetal bovine serum (FBS) (Invitrogen), and grown in a humidified cell culture incubator (37°C, 5% CO_2_). The cells were trypsinized, diluted to 10^5^ cells/mL, and then distributed onto 96-well cell-culture-treated plates. 24 hours later, the cells received toxin and were diluted in DMEM +10% FBS (100 µl/well final volume) in one of two formulations. In the first, cells were incubated at 4°C for 1 hour, 100 µl/well toxin-containing media was removed and replaced by fresh media, and cells were returned to the incubator to grow for 24 hours. In the second, toxin was simply added to the cells and incubated for 24 hours. The cells were then lysed using 100 µl/well 1/5 dilution of CellTitre-Glo reagent (Promega), and luminescence was measured using a Victor II plate reader. A 50% cytotoxic dose (CD_50_) represents the amount of toxin necessary to kill 50% of the attached monolayer of cells in the wells. CD_50_’s were approximated by plotting three points within the linear portion of the graph, and solving for 50% (0.5). This was typically 2.72^(0.5−a)/b^ for semi-log, where a and b are determined by the plot. All photographs were taken using a Leica DM IL microscope at 200x magnification.

### pH and Heat Treatments

To pH treat Stx2f and Stx2a, 500 ng/mL toxin was diluted in NaOAc buffer (250 mM) at various pH values and allowed to incubate at RT for 1 hour. To heat treat Stx2f and Stx2a, 500 ng/mL toxin was incubated in NaOAc buffer at indicated temperatures for 1 hour. After treatment, the mixture was diluted 100-fold with DMEM/FBS culture media, and incubated 100 µl/well with Vero cells for 1 hour at 4°C, and then the media was replaced with 100 µl fresh DMEM/FBS for 24 hours. Cells were lysed and luminescence measured using a 1/5 dilution of 100 µl/well CellTitreGlo reagent and a Victor II plate reader, as above. Treating Vero cells with a 2.5 mM, pH 1.5 NaOAc in DMEM/FBS control had a negligible toxicity (data not shown).

## Results

### Expression and Purification of the Stx2f Subtype

**Figure 1:**
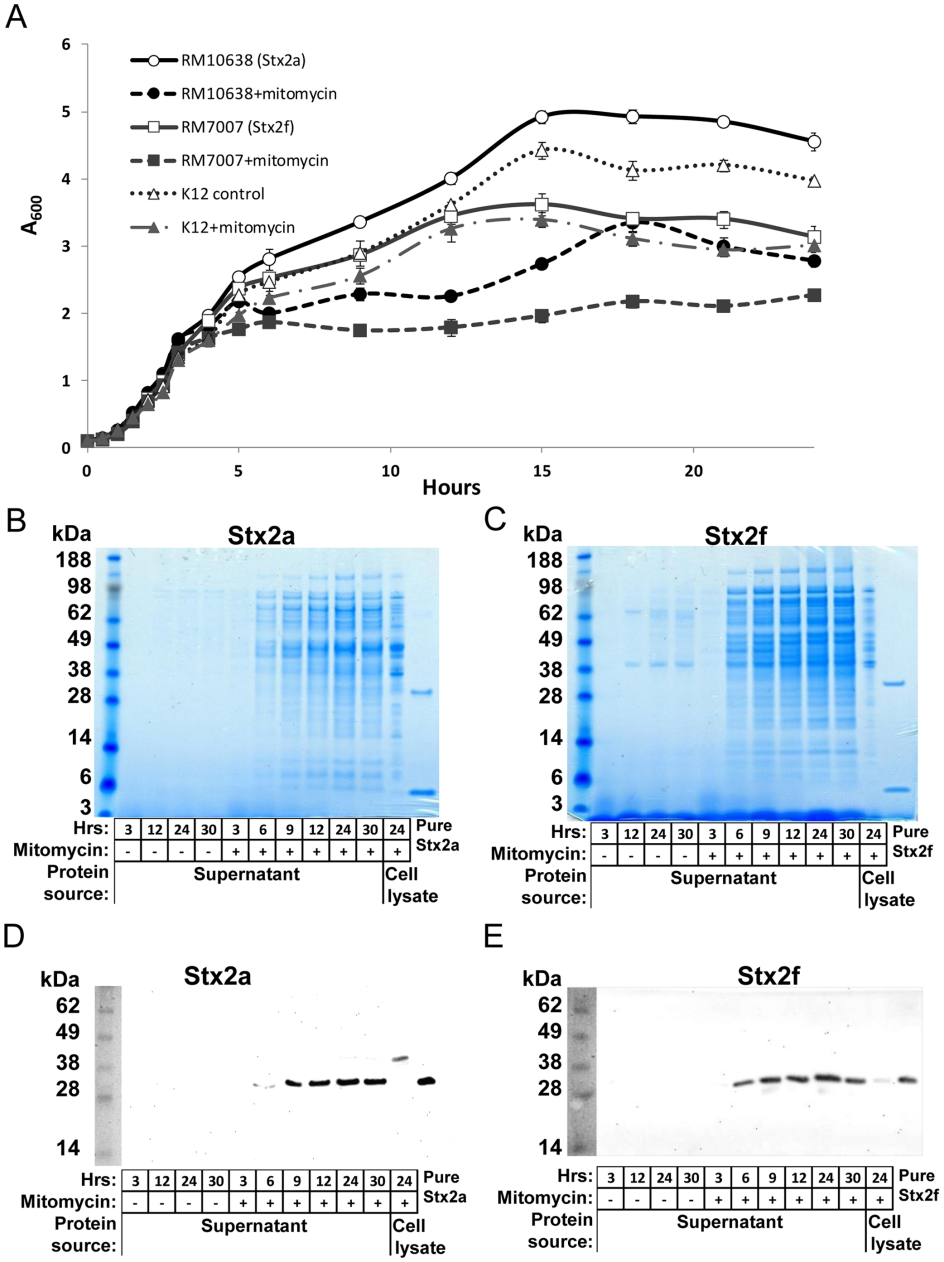
Induction of Stx2a and Stx2f production. A. Growth curves for Stx2a-expressing (RM10638) and Stx2f-expressing (RM7007) strains, with and without 50 ng/mL mitomycin C, measured by absorbance at 600 nm. The K12 strain of *E. coli* is used as a control. B. Timecourse of protein release into the media by a Stx2a-expressing strain (RM10638), analyzed by SDS-PAGE and Coomassie staining. Each lane contains 13 µL of media or cell lysate. Lane 1 to 4: no mitomycin C added. Lane 5–10: mitomycin C was added at 50 ng/mL. Lane 11: cell lysate with 50 ng/mL mitomycin C. Lane 12: Stx2a pure protein control (0.5 µg). C. Timecourse of phage-induced lysis of a Stx2f-expressing strain (RM7007), analyzed by SDS-PAGE and Coomassie staining. Lane designations are as in B), with Stx2f being used as the protein control. D. Stx2a production by RM10638, detected by Western blot with an anti-Stx2a rabbit polyclonal antibody. Each lane contains 2 uLs of media or cell lysate. Mitomycin C treatment follows that of B) and C). Protein control lane contained 0.01 µg Stx2a. E. Stx2f production by RM7007, detected by Western blot with an anti-Stx2f mouse polyclonal antibody. Amount of media or cell lysate and mitomycin C treatment follows that of D). Protein control lane contained 0.01 µg Stx2f.

*E. coli* strains (Table 1) expressing Stx2f or Stx2a share similar growth kinetics (Figure 1A), although the Stx2a strain grows more robustly and reaches a higher maximum optical density (OD). Production of toxins and lysis of cells in both strains are greatly increased by treatment with mitomycin C (Figure 1B, 1C, respectively). Maximal expression and liberation of the Stx2f toxin into the culture media was achieved after 24 hours of bacterial growth with 50 ng/mL mitomycin C (Figure 1C). This corresponds to stationary phase in these cultures, and is similar to the expression of Stx2a. The quantity of Stx in the media was vastly greater than in the cell lysate (Figure 1C), thus Stx2f and Stx2a were purified exclusively from filtered media.

**Figure 2:**
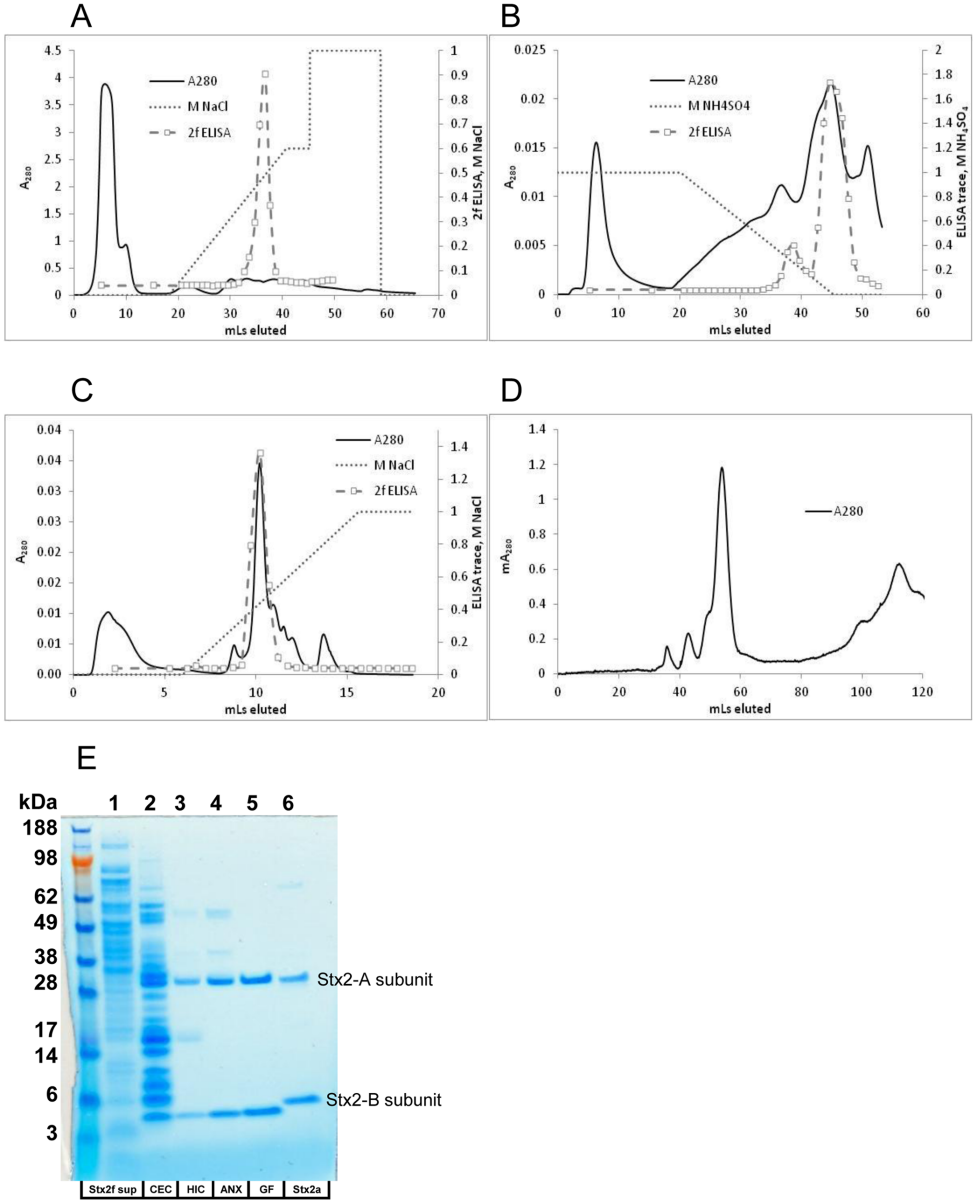
Purification of Stx2f. A. Cation exchange chromatography (CEC) of Stx2f-containing media. Media was fixed to pH 4 with glacial acetic acid then loaded onto the column. B. Hydrophobic interaction chromatography (HIC) of top four Stx2f-containing CEC fractions. C. Anion exchange chromatography (AEC) of top four Stx2f-containing HIC fractions. D. Gel filtration (GF) of Stx2f-containing top three AEC fractions. E. Purification procedure, resulting in pure Stx2f. Lane 1: Stx2f culture media, filtered. Lane 2: Top four combined fractions from CEC. Lane 3: Top four combined fractions from HIC. Lane 4: Top three combined fractions from AEC. Lane 5: Top two combined fractions from GF, pure Stx2f. Lane 6: Stx2a protein control. 2 µg of protein (measured by BCA assay) were loaded into lanes 1 and 2, and 0.5 µg of protein were loaded into lanes 3–6.

Purification schemes previously developed for Stx2a involve multiple steps using size and charge-based chromatography [31] or antibody affinity columns [26], [32]. Since no commercially available monoclonal antibodies bind Stx2f with high affinity, we focused on developing a semi-automated column-based purification regimen using an Äkta FPLC (GE Biosciences). In order to obtain pure Stx2f, a four-step protocol was established: media-pH-fixed cation exchange chromatography (CEC) (Figure 2A), followed by hydrophobic interaction chromatography (HIC) (Figure 2B), then anion exchange chromatography (AEC) (Figure 2C), and finally gel filtration (GF) (Figure 2D). Elution of the toxin was monitored by ELISA using an anti-Stx2f A subunit mouse polyclonal antibody developed in this study. After the final purification step, GF, a preparation that was purified 4524-fold with a recovery of 2% (as measured by Vero cell toxicity) was obtained (Table 2) (Figure S1). This purification scheme produced 5.2 µg of purified Stx2f from 450 mL of bacterial culture supernatant (Table 2).

**Table 2:** Purification yields from each of the four steps in Stx2f purification, as measured by CD_50_.

Sample	Total mL	% recovery	Fold purification	CD_50_ pg/well
2f sup	450	100%	1	7690.3±622
CEC	3	46%	405	19.0±2.1
HIC	3	11%	1114	6.9±1.5
AEC	0.4	3%	2136	3.6±1.1
GF (pure)	0.2	2%	4524	1.7±0.5

### Biochemical Properties of the Stx2f Subtype

The purity and molecular weights for Stx2f subunits after the GF step were analyzed using SDS-PAGE followed by Coomassie staining. Only two bands were visible on the gel (Figure 2E, lane 5). The upper band has a molecular weight similar to the A subunit of the Stx2a (as indicated in Figure 2E, lane 6), but the MW of the lower band is clearly smaller than the B subunit of the Stx2a. Based on the estimation using the ExPASy Compute pI/MW tool, the MWs of Stx2f and Stx2a are very similar (Stx2a: 35.8 kDa for the A subunit, 9.9 kDa for the B subunit; Stx2f: 35.6 kDa for A, 9.6 kDa for B). It’s not clear why the Stx2f B subunit appears smaller on the SDS-PAGE. The estimated isoelectric point (pI), using the same tool, for Stx2a and Stx2f varies dramatically (Stx2a pI: 6.3; Stx2f pI: 9.1). The extinction coefficient of our purified Stx2f toxin protein was 0.60 mL mg^−1^ cm^−1^ at 280 nm.

### Receptor-binding of Stx2f

**Figure 3:**
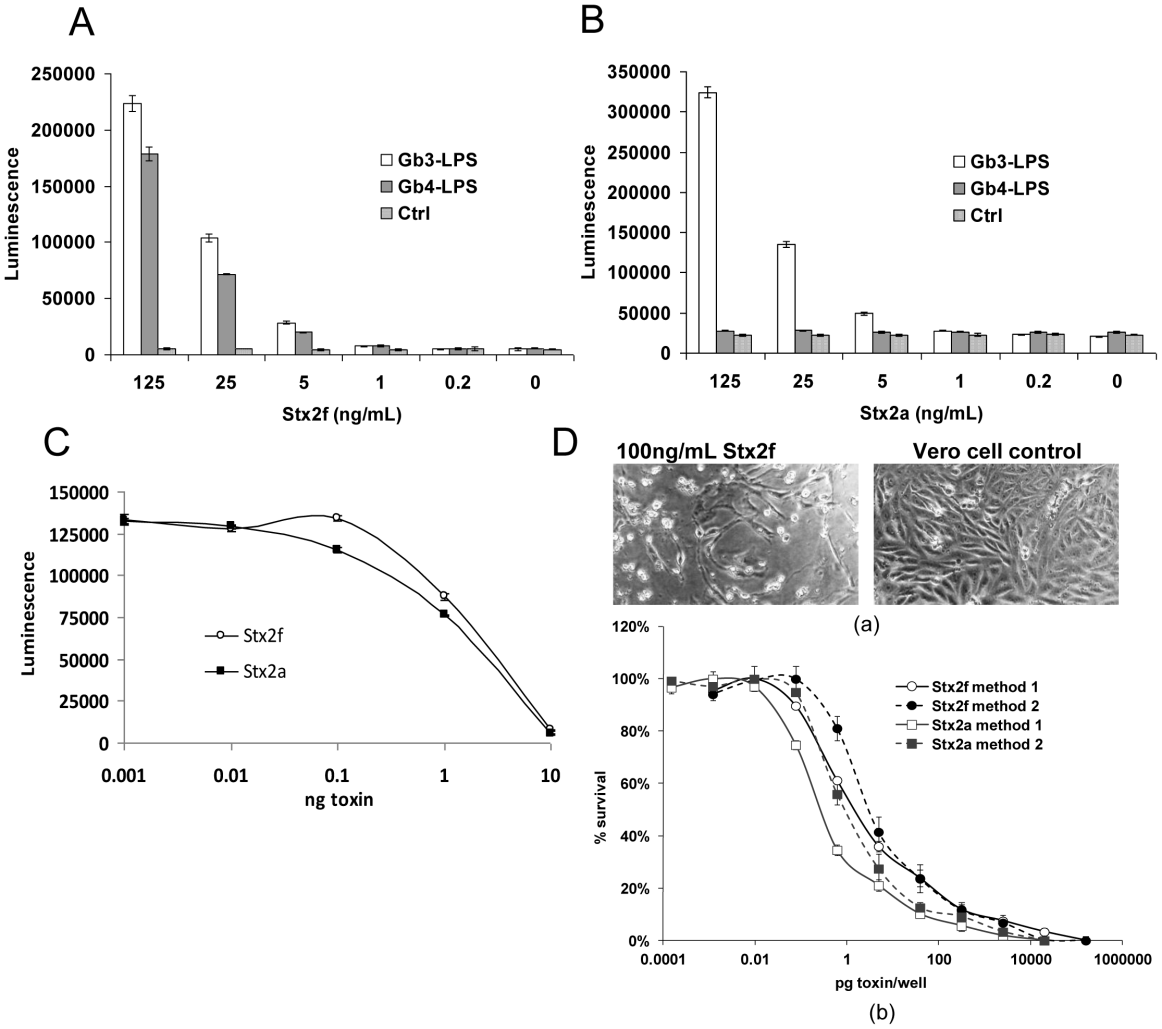
Biochemical properties and cell toxicity of Stx2f. A. Gb3-LPS and Gb4-LPS binding for Stx2f. Binding was measured in an ELISA using *E. coli* cells expressing Gb3-LPS (Gb3 mimic) or Gb4-LPS (Gb4 mimic), or control cells. A representative experiment is shown (N = 4). B. Gb3-LPS and Gb4-LPS binding assay for Stx2a. Binding was measured as in (A). A representative experiment is shown (N = 3). C. *In vitro* translation is inhibited by Stx2f and Stx2a to similar degrees in a rabbit reticulocyte lysate assay. A representative experiment is shown (N = 3). D. Stx2f is toxic to Vero cells in culture, but Stx2a is more toxic. Method of treatment (overnight incubation with toxin: Method 1; treatment at 4°C [1 hour] followed by media exchange: Method 2) is important when calculating CD_50_ values. Stx2a is more toxic than Stx2f using both methods: by 5-fold using Method 1 (0.3 and 1.6 pg/well, respectively), and 3-fold using Method 2 (1.1 and 3.4 pg/well, respectively) (N = 4).

To compare the interactions of Stx2f with Gb3Cer and Gb4Cer receptors, binding of purified Stx2f to cells globotriose- or globotetraose-lipopolysaccharide (Gb3-LPS or Gb4-LPS) on the surface was measured by “sandwich” ELISA. *E. coli* cells expressing Gb3-LPS or Gb4-LPS were immobilized on the 96-well plates and binding of Stx2f was observed using the mouse polyclonal antibody against Stx2f for detection. It was found that Stx2f bound to both Gb3-LPS and Gb4-LPS-containing *E. coli* cells. Although Stx2f has a mild preference for Gb3-LPS (Figure 3A), it exhibited relatively strong binding to Gb4-LPS cells when compared with Stx2a, which is known to bind Gb4Cer weakly in other assays [33]. In our experimental conditions, Stx2a did not bind Gb4-LPS cells, and exhibited an overwhelming binding preference toward Gb3-LPS cells (Figure 3B).

### Enzymatic Activity and Cytotoxicity of Stx2f

Much of the active site of Stx2f is identical to that of Stx2a, including the N75, Y77, E167, R170, and R176 positions that, when mutated, eliminated toxicity [34]. Additionally, Stx2f-containing media inhibited translation in an *in vitro* assay [35]. We therefore assumed that Stx2f, like other subtypes of Stx2, possesses an exposed catalytic site for *N-*glycosidase activity. An *in vitro* translation assay conducted on rabbit reticulocyte lysate (Promega) confirmed that intact (A+B pentamer) purified Stx2f is capable of inactivating ribosomes, resulting in the halting of protein synthesis (Figure 3C). The midpoint concentration (IC_50_) for inhibition of translation was 3.5 µg/mL, a little higher than that of Stx2a (2.5 µg/mL). Although purified Stx2f effectively kills Vero cells (Figure 3D-a), it is less toxic than Stx2a (Figure 3D-b). Vero cells were treated by two different methods and the 50% cytotoxic dose (CD_50_) of Stx2f was shown to be 3- or 5-fold higher than that of Stx2a, indicating that Stx2f is considerable less toxic compared to Stx2a (Table 3). The difference between these two methods is (1) adding toxin to Vero cells and incubating the cells at 37°C overnight or (2) adding toxin, incubating the cells with the toxin for an hour at 4°C, and replacing unbound toxins with fresh medium, then incubating the cells at 37°C overnight. We observed that these two methods are not equivalent. Method (1) was as much as 5 times more sensitive than method (2). Dissimilar CD_50_ values have been reported for Stx2a [36], [37], these differences may be due to the Vero cell treatment method, the cell viability assay, the original purity of the toxin, and the purification protocol. For these reasons, comparisons to absolute values are valid within a study, but not necessarily across studies.

**Table 3:** Calculated CD_50_’s for Stx2f and Stx2a by two different treatment methods.

Toxin	Method	CD_50_ pg/well
Stx2f	1^a^	1.6±0.2
Stx2f	2^b^	3.4±0.4
Stx2a	1	0.3±0.03
Stx2a	2	1.1±0.3

a Sterile cell-free media added directly to Vero cells.

b Sterile cell-free media added to Vero cells, incubated for 1 hour at 4°C, removed, and replaced with DMEM +10% FBS.

### Stability of the Stx2f Subtype

**Figure 4:**
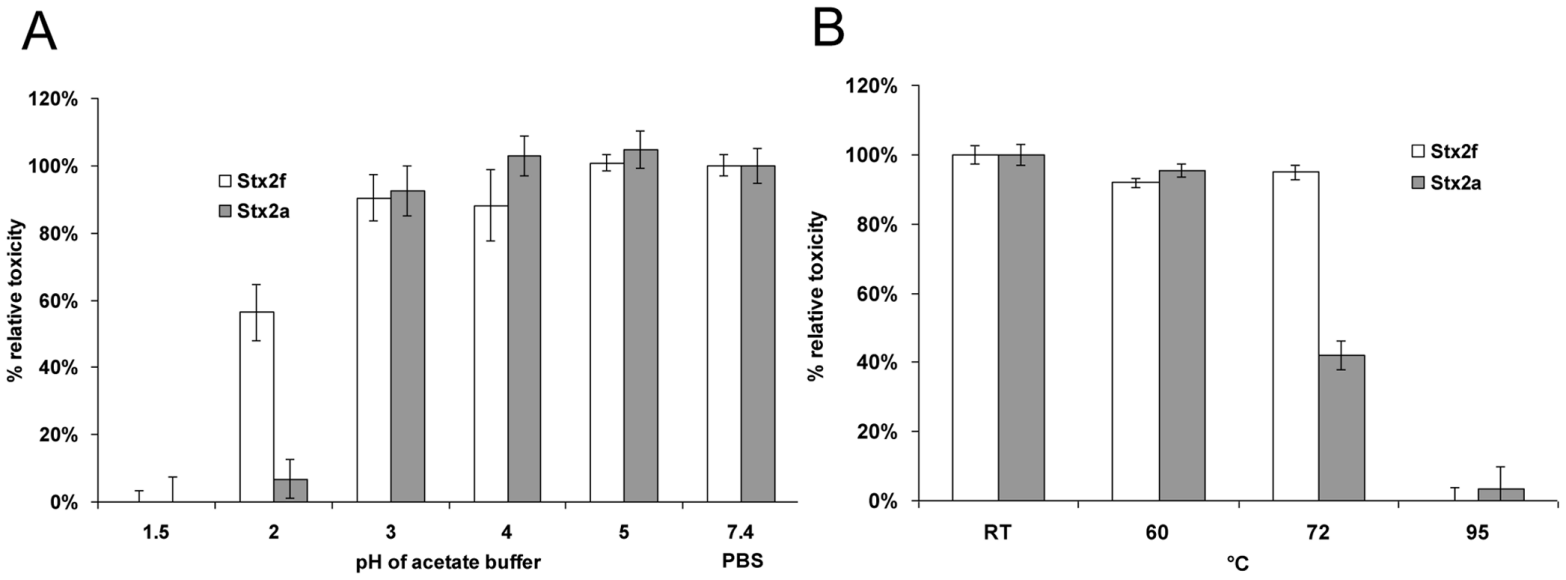
Stability of the Stx2f subtype. A. Toxicity of Stx2f and Stx2a after a one hour incubation under the indicated low pH conditions. Toxins were applied at a 5 ng/mL concentration. A representative experiment is shown (N = 5). B. Toxicity of Stx2f and Stx2a after a one hour incubation at the indicated elevated temperatures. Toxins were applied at a 5 ng/mL concentration. A representative experiment is shown (N = 3).

Acetate produced by commensal microorganisms may be responsible for generating a low intestinal pH that is inhibitory to Stxs as well as Stx expression [38]. Therefore, we investigated the effects of low pH in acetate buffer upon Stx2a and Stx2f toxicity in Vero cells. After an hour-long incubation in 250 mM sodium acetate at pH 1.5, the toxicity of Stx2f and Stx2a was completely eliminated. However, after a pH 2 treatment, Stx2a had lost the most of its toxicity, whereas Stx2f remained highly potent (Figure 4A, Figure S2). Since Stx2f is more stable at low pH, we postulated that Stx2f might be more thermo-tolerant as well. Although incubation at 95°C thoroughly inactivated both Stx2f and Stx2a, a 72°C incubation rendered Stx2f more toxic than Stx2a, by a considerable margin (Figure 4B). This information suggests that, while Stx2f is less toxic than Stx2a in cell culture, it generally appears to be more stable, both to low pH and heat.

## Discussion

STECs and Stxs represent a serious and continually evolving public health concern. STECs are responsible for some of the most expansive and deadly food-borne disease outbreaks in recent history, and research into detecting, characterizing, and neutralizing Stxs, particularly Stx2s, is a key to preventing and treating future outbreaks. This is a complicated issue, however, STEC serotypes and Stxs themselves are highly diverse. All seven of the predominant Stx2 subtypes (Stx2a to 2g) have been associated with human illness [39], and all are detectable by PCR and immunoassays, but each detection method seems to have certain subtype specificities. In particular, Stx2f is undetectable by all but a few commercial immunoassays (VTEC-RPLA assay being one of the exceptions) [6], and has evaded most attempts at characterization thus far, since purified Stx2f has not been demonstrated. In this study, we detail a purification scheme for Stx2f. Using pure Stx2f toxin, we assess its preferred binding ligand, catalytic activity, toxicity to Vero cells, and stability in environments it may encounter in food preparation or during its passage to the intestine.

Although novel, the purification procedure for highly pure Stx2f detailed here is somewhat involved and generates a low yield of toxin. However, obtaining pure toxin greatly simplifies production of toxin-specific monoclonal antibodies. Once high-affinity monoclonal antibodies to Stx2f are available, a one or two-step affinity purification should become routine and robust, both for ease and yield. In order to monitor the Stx2f toxin while performing its purification, Stx2f mouse anti-serum was prepared. The antigen used was a His-tagged version of the Stx2f A subunit, presumed to be the more epitope-rich subunit, and the presence of the 6xHis tag made it easy to purify. For future polyclonal antibody preparations, however, a catalytically inactive recombinant toxoid complex such as the E167Q mutant would make a better antigen [26]. Using this antigen, which assembles as a non-toxic AB_5_ toxoid, both the A and B subunits should be detectable. If a Stx2f toxoid was available for comparison, it would also be interesting to note if the B subunit of the toxoid is smaller than that of Stx2a. This phenomenon (Stx2f B being smaller than Stx2a B, Figure 2E) is difficult to explain, but the purified Stx2f B subunit is clearly functional (it effectively kills Vero cells in culture). The Stx2f B subunit may be subjected to unintended proteolysis sometime during the purification process. This could be determined by mass spectrometry analysis of the purified Stx2f (in preparation).

Despite exhibiting only a 71% and 82% (A and B subunit, respectively) amino acid sequence identity to Stx2a [35], Stx2f has been shown in this and previous studies to effectively kill Vero cells in culture [24], and is very similar to Stx2a in terms of catalytic activity (Figure 3C). However, unlike Stx2a, the presence of Stx2f in *E. coli* is not currently associated with HUS. The low frequency of Stx2f isolates in human disease may be due to the possibility that the Stx2f phage has not yet established itself in an *E. coli* strain that is capable of human pathogenicity. If this is the case, it may be only a matter of time before Stx2f isolates become serious human pathogens. Although we don’t know how toxic pure Stx2f is *in vivo*, Stx2f is less toxic to Vero cells than Stx2a. Since the catalytic activities of Stx2f and Stx2a appear to be equivalent, differences in receptor preference, receptor affinity, or stability between the Stx2a and Stx2f subtypes may be responsible for the difference in toxicity. Specificity or affinity of the receptor appears to be a determinant of toxicity [40]. The preferred receptor for Stx2e is Gb4, although it also binds well to Gb3 [33], and it is thought that this is why Stx2e, which can be deadly to pigs, usually only causes mild gastroenteritis in humans. Here, we report that Stx2f has affinity toward both Gb3-LPS and Gb4-LPS, with a slight preference toward Gb3-LPS (in the assays we conducted). This is not surprising, since the B subunits of Stx2e and Stx2f are nearly identical, and differ by only two amino acids. The B subunit is responsible not only for receptor binding, but also receptor preference, as illustrated by Stx2e mutations. When the Q64E/K66Q double mutation was incorporated into the Stx2e B subunit, its receptor preference changed from Gb3/Gb4 to Gb3 [41]. Stx2f, like Stx2e, possesses Q64/K66, and binds to both Gb3-LPS and Gb4-LPS. In any case, it is possible that Stx2f is not as potent as Stx2a in Vero cells due to a “dilution” among both Gb3 and Gb4 receptors, though the mechanism for this is unclear. Another possibility is that, with its promiscuous binding, Stx2f is more evenly distributed across the cell surface and internalized in vesicles at a lower concentration, and that a high concentration of Stx2 in vesicles would result in a strong pulse of toxin, and greater toxicity. Of course, the reduced toxicity of Stx2f could also be mediated by reduced receptor binding, internalization, or cytoplasmic release, among other factors. A-B subunit chimeras between Stx2f and Stx2a could be helpful in determining which subunit is responsible for the attenuated toxicity. A comparison between mouse toxicity and Vero cell toxicity could be very revealing as well. Certainly, these studies will be a focus of future work.

Stability of Stxs also appears to differ among characterized subtypes. Stx2f, despite its low relative toxicity compared to Stx2a, is more stable to both low pH and thermal treatment. Pure Stx2f maintains its toxicity after a pH 2 or 72°C treatment significantly better than Stx2a (Figure 4A and 4B). Our previous results indicate that the thermal stability of Stx2a, especially its catalytic activity, could change depending on its origin and purity [29]. However, the Stx2a and Stx2f were purified using a similar protocol to a similar purity in this study, suggesting that Stx2f may possess a more thermally stable molecular structure. Still, more experimentation would be necessary to determine whether the lower toxicity of 72°C-treated Stx2a is actually due to reduced catalytic activity of the A subunit or other factors. The relatively low measured thermal stability of Stx2a toxin activity may also be due to deactivation of the B subunit for cell surface receptor binding. It is possible that the Stx2f AB_5_ complex itself might be more stable, and requires harsher conditions to cause it to fall apart. Fascinatingly, a similar phenomenon is evident for a pH 2 treatment (Figure 4A), where Stx2f loses only half its toxicity and Stx2a is almost completely non-toxic. It has been shown previously that Stx2a remains stable until below pH 3 when incubated with pH-fixed PBS, which is consistent with our results [30]. We have postulated in this study that acetate produced by intestinal microbiota might not only inhibit Stx2 toxin expression [38] but also inactive the toxin itself. At pH 2 it can inhibit Stx2a, and at pH 1.5 it can even inhibit Stx2f, but the acetate generated by commensal bacteria such as *Lactobacillus* and *Bifidobacterium* is unlikely to lower intestinal pH to that level: *Bifidobacterium* raises the intestinal acetate concentration to 56 mM and lowers the intestinal pH to only 6.75 [42]. pH 2 is close to the pH of gastric acids, and ominously suggests that Stx2f could survive the journey from mouth to intestine better than Stx2a. However, Stxs are usually produced from the intestine, so the pH stability of Stx2f may be irrelevant at the physiological level of human pathogenicity. Other studies have implied that Stx evolved for defense against protozoan predation, for which resistance to highly acidic endolysosomal vacuoles would be an invaluable asset [43]. This would provide a reason for the general acid stability of Stx2a and Stx2f and suggests that *E. coli* strains harboring Stx2a and Stx2f might be exposed to different sets of protozoan predators.

Although common in a ubiquitous animal vector (pigeons), Stx2f-encoding STEC strains are not yet considered a major health concern due to the lack of severity and rarity of infections. However, since most Stx immunoassays are poor at detecting Stx2f, it is becoming clearer that Stx2f infections are more common than we realize [25]. Although less toxic than Stx2a in Vero cells, Stx2f does appear to be generally more stable, and may persist more readily during food preparation. This study should highlight the need for a robust detection system for Stx2f, something that is made more attainable by purified Stx2f toxin, so foodstuffs contaminated with Stx2f-containing STEC can be readily identified and removed.

## Supporting Information

Figure S1 **Vero cell toxicity curves of purification steps used to calculate the CD_50_ values in** **Table 2** **.** The X-axis is total nanograms of protein added per well.
(TIF)

Figure S2 **Vero cells are more sensitive to pH 2-treated Stx2f than to pH 2-treated Stx2a.** Photographs are of Vero cells used in Figure 4A.
(TIF)
