# Supplementary figures and images for: Purification and Characterization of Shiga Toxin 2f, an Immunologically Unrelated Subtype of Shiga Toxin 2
Source: PLoS One. 2013 Mar 26;8(3):e59760. doi: 10.1371/journal.pone.0059760 (PMC3608586; doi:10.1371/journal.pone.0059760)

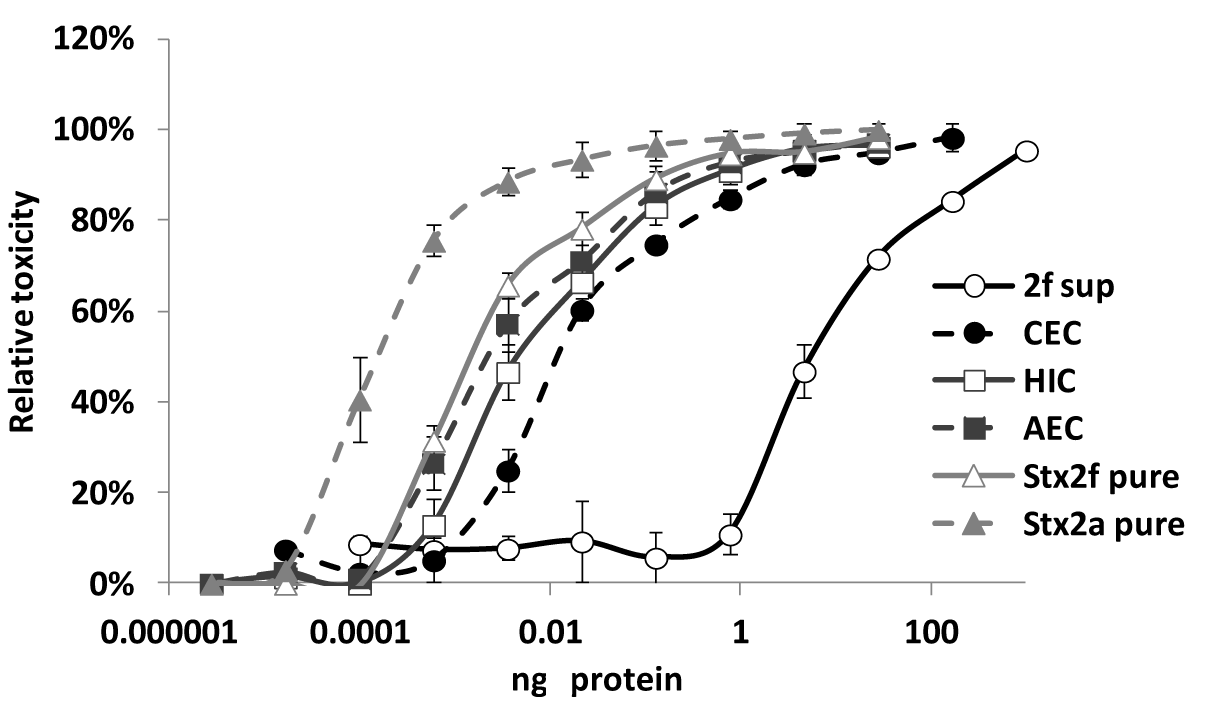

Supplement: Figure S1 — Vero cell toxicity curves of purification steps used to calculate the CD50 values in Table 2 . The X-axis is total nanograms of protein added per well. (TIF) [file pone.0059760.s001.tif]

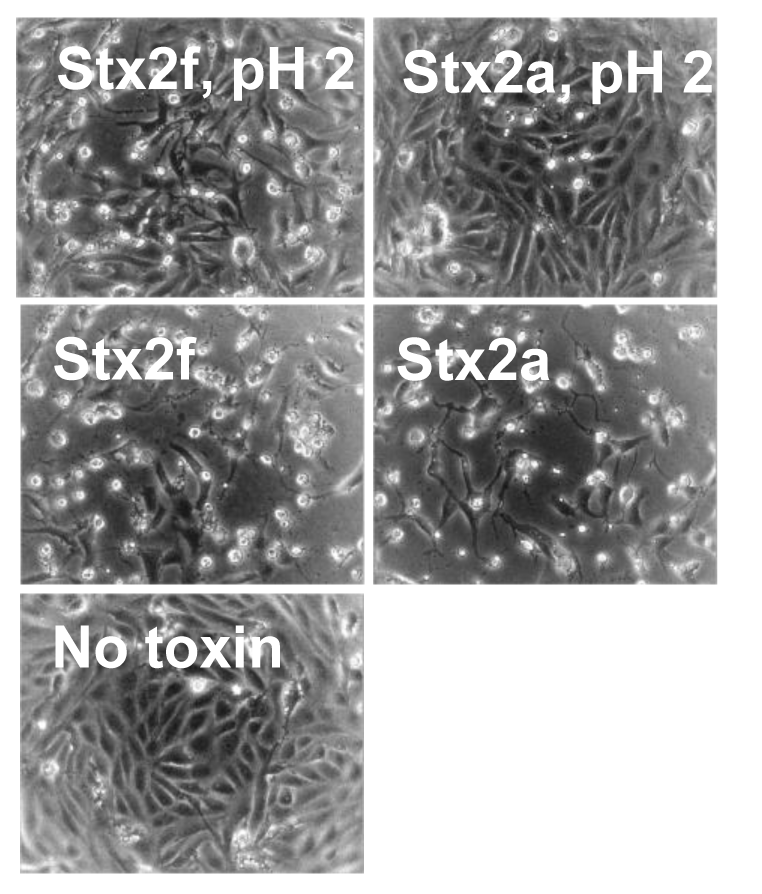

Supplement: Figure S2 — Vero cells are more sensitive to pH 2-treated Stx2f than to pH 2-treated Stx2a. Photographs are of Vero cells used in Figure 4A. (TIF) [file pone.0059760.s002.tif]
